# Importance of KIM-1 and MCP-1 in Determining the Leptospirosis-Associated AKI: A Sri Lankan Study

**DOI:** 10.1155/2021/1752904

**Published:** 2021-06-02

**Authors:** Thilini Nisansala, Manjula Weerasekera, Nilantha Ranasinghe, Chamil Marasinghe, Chandika Gamage, Neluka Fernando, Chinthika Gunasekara

**Affiliations:** ^1^Department of Microbiology, Faculty of Medical Sciences, University of Sri Jayewardenepura, Sri Lanka; ^2^Base Hospital, Panadura, Sri Lanka; ^3^Department of Medicine, Faculty of Medical Sciences, University of Sri Jayewardenepura, Sri Lanka; ^4^Department of Microbiology, Faculty of Medicine, University of Peradeniya, Sri Lanka

## Abstract

**Background:**

Acute kidney injury (AKI) is one of most prevalent and serious complications of leptospirosis, a prevalent zoonotic disease in tropical countries. Prompt diagnosis of the leptospirosis-associated AKI is a challenge as there are no proper diagnostic tools that can identify patients in the early stage. Kidney injury molecule-1 (KIM-1) and monocyte chemoattractant protein-1 (MCP-1) are widely used novel AKI biomarkers that are studied in various disease conditions with AKI, but not in leptospirosis. Thus, this study is aimed at seeking the importance of KIM-1 and MCP-1 in determining the leptospirosis-associated AKI.

**Methods:**

Leptospirosis-suspected patients who were admitted to medical wards of two selected hospitals in the Western province of Sri Lanka were recruited. Leptospirosis was confirmed by three diagnostic tests: PCR, MAT, and culture, and the status of AKI was determined by Kidney Disease Improving Global Outcomes (KDIGO) criteria.

**Results:**

Of 170 leptospirosis-suspected patients, 79 were leptospirosis confirmed, and among them, 24.05% of patients were diagnosed to have AKI according to KDIGO criteria. Median serum KIM-1 (*p* < 0.0001), urine KIM-1 (0.0053), serum MCP-1 (0.0080), and urine MCP-1 (0.0019) levels in those developing AKI were significantly higher than in patients not developing AKI. The biomarker levels associated with leptospirosis AKI had AUC-ROC of 0.8565, 0.7292, 0.7024, and 0.7282 for serum KIM-1, urine KIM-1, serum MCP-1, and urine MCP-1, respectively.

**Conclusion:**

This study revealed serum KIM-1 as a promising marker for leptospirosis-associated AKI among the tested biomarkers. Thus, further validation is recommended with a larger study group.

## 1. Introduction

Acute kidney injury (AKI) in leptospirosis is one of the major complications causing high morbidity and mortality among the spectra of complications associated with leptospirosis [1]. Renal involvement in leptospirosis may range from uncomplicated mild symptoms characterized by slight proteinuria and urinary sediment deviations to severe AKI requiring dialysis [2]. Leptospirosis-associated AKI is a rare incidence in developed countries; nevertheless, in tropical countries, leptospirosis is one of the most common etiologies of AKI [3]. Leptospirosis was an etiology for 13% of AKI cases in India and more than 20% in Thailand and Singapore [4, 5]. In a recent study done in the Western and Southern provinces of Sri Lanka, AKI was reported in 35.7% of patients with confirmed leptospirosis while another study done in the North Central province and Central province in Sri Lanka reported 22% and 15% of patients with confirmed leptospirosis, respectively, highlighting its wide distribution in the country [6–8]. These studies done in Sri Lanka emphasize AKI as a major complication of leptospirosis in the country. It is important to identify patients likely to develop AKI during the course of infection, in order to prevent complications and mortality.

Although pathogenesis of AKI in leptospirosis has not been fully elucidated, studies conducted with this regard suggest immune-mediated damage [9–12]. While renal impairment in leptospirosis is characterized by the presence of interstitial nephritis and tubular damage [13], direct nephrotoxic action of the *Leptospira* and hemodynamic alterations as well as rhabdomyolysis is also considered as contributory factors [11, 14].

Complications of leptospirosis can be prevented by prompt diagnosis of the disease, thereby starting early antibiotic treatment and proper supportive therapy. However, early diagnosis of leptospirosis and early detection of renal complications are major challenges in the management of these patients [9, 15]. Serum creatinine levels are routinely used in the identification of patients developing renal complications [16]. Many criteria assessing AKI such as the Risk, Injury, Failure, Loss, End-stage renal disease (RIFLE) as well as its modification Acute Kidney Injury Network (AKIN) and Kidney Disease Improving Global Outcomes (KDIGO) guideline have been used by groups to diagnose AKI [17–19]. These criteria rely heavily upon the level of serum creatinine [16]. However, it is widely accepted that serum creatinine levels do not rise until around 50% of kidney function is lost [20]. In Sri Lanka, serum creatinine levels are a major consideration in the diagnosis of AKI due to unavailability of more appropriate and easily available markers in the local setting.

Kidney injury molecule-1 (KIM-1) and monocyte chemoattractant protein-1 (MCP-1) are among the serum novel biomarkers with emerging roles as potential early diagnostic markers expressed in AKI [21–23]. KIM-1 is involved in epithelial cell regeneration and phagocytosis of dead cells in the tubular lumen [24]. MCP-1 is a potent chemotactic factor for monocytes and has shown promising results as an early diagnostic marker in AKI in a murine model [25]. This study describes the levels of KIM-1 and MCP-1 in leptospirosis-confirmed patients. Further, we aimed to determine the usefulness of these two novel biomarkers as a diagnostic tool to predict AKI in leptospirosis.

## 2. Methods

### 2.1. Study Design and Study Sample

This was a prospective hospital-based study conducted in a tertiary care and base hospital in the Western province of Sri Lanka between January and December 2017. Patients presenting with clinically suspected leptospirosis admitted to the medical wards were recruited for the study following informed written consent. The inclusion criteria of this study were based on “suspected case definition” given in Communicable Disease Epidemiology Profile Sri Lanka, World Health Organization [26]. Patients less than 18 years of age were excluded from the study. The study population consisted of patients with fever between days 01 and 16 on admission.

Five milliliters of blood was collected following standard procedures and aliquoted to a plain tube for serum separation and to an ethylenediaminetetraacetic acid (EDTA) tube for DNA extraction. A urine sample was collected into a sterile wide mouth container. All samples were collected on the day of admission of the patient and transported at 4°C to the laboratory. Serum was separated and aliquoted, and all specimens were stored at -80°C until further processed.

### 2.2. Data Collection

All data including sociodemographic profile, clinical features, and risk factors were collected using a pretested interviewer-administered questionnaire. Clinical data and basic laboratory findings (complete blood count, serum electrolytes, renal profile, and liver profile) were collected from the Bed Head Ticket (BHT) of each patient. The serum creatinine levels were determined using a fully automated biochemical analyzer following the manufacturer's instructions. The outcome and complications of each patient were recorded.

### 2.3. Laboratory Confirmation of Leptospirosis

Microscopic agglutination test (MAT), culture isolation, and real-time polymerase chain reaction (PCR) were used to confirm leptospirosis in patients [27–29].

#### 2.3.1. Microscopic Agglutination Test (MAT)

MAT was carried out using a panel of 15 reference *Leptospira* strains at the Medical Research Institute, Sri Lanka, which is the national reference laboratory (28).

#### 2.3.2. Real-Time PCR for Detection of *Leptospira*

DNA was extracted from EDTA whole blood specimens using a QIAamp DNA blood mini kit (QIAGEN GmbH, Germany) following the manufacturer's instructions. Real-time PCR (Bio-Rad CFX96™ (Bio-Rad, USA)) based on EvaGreen technology was performed to detect pathogenic *Leptospira* spp., using secYIVF and secYIV [27].

#### 2.3.3. Culture of *Leptospira*

*Leptospira* was isolated from patients' blood by inoculating into semisolid EMJH medium and confirmed by *Leptospira* flaB PCR as previously described [29, 30].

A patient was considered as “leptospirosis confirmed” if positive by one of the above three tests while patients who were enrolled into the study due to clinical suspicion of leptospirosis but were negative for any of these tests were considered as “leptospirosis unconfirmed.” The KDIGO AKI guideline was used to classify the AKI status of patients [19].

### 2.4. Enzyme-Linked Immunosorbent Assay (ELISA) for Renal Markers KIM-1 and MCP-1

Both serum and urine KIM-1 (R&D, Minneapolis, USA) and MCP-1 (BioLegend, San Diego, California, USA) expression levels were measured by ELISA following the manufacturers' instructions. ELISAs were validated by intra-run precision, inter-run precision, and dilution verification. Standard curves were generated following the manufacturer's instructions. KIM-1 and MCP-1 standards were prepared by reconstitution of the standard vials in the provided diluents. Serum and urine specimens for MCP-1 ELISA were used at the original concentration while samples were diluted by half for KIM-1 ELISAs. All standards and specimens were carried out in duplicate. Mean absorbance values were calculated, and data were analyzed using GraphPad Prism version 6.05 (GraphPad Software Inc.) to determine the level of biomarkers.

### 2.5. Statistical Analysis

Categorical variables were expressed as proportions and compared using a two-sample proportion test. Continuous variables were expressed as mean ± standard deviation (SD) (data with normal distribution) and compared using the unpaired *t*-test or were expressed as median ± interquartile range (IQR) (data with skewed distribution) and compared using Mann-Whitney *U* test, as appropriate. Spearman's coefficient test was performed to determine the correlation between serum creatinine and biomarkers. Receiver operating characteristic (ROC) curves were generated, and the area under the curve (AUC) was calculated to assess the predictive ability of each biomarker. Data were analyzed using Statistical Package for the Social Sciences (SPSS version 20) and GraphPad Prism version 6.05. *p* values < 0.05 were considered as statistically significant.

## 3. Results

### 3.1. Patients' Clinical and Demographic Characteristics

**Figure 1:**
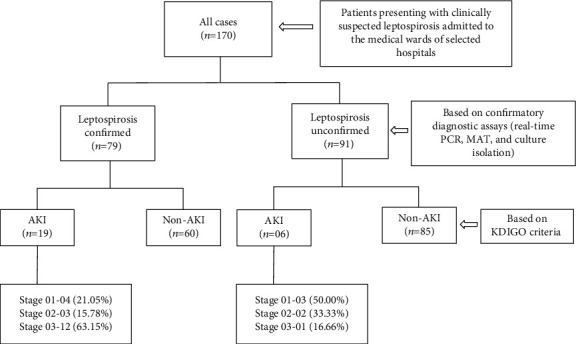
Classification of the study population.

One hundred and seventy clinically suspected leptospirosis patients were recruited during the study period. Leptospirosis was confirmed in 79 patients (46.47%) by a positive MAT (*n* = 44), PCR (*n* = 49), or culture (*n* = 2) (Figure 1). Out of the 79 confirmed patients, 69 (87.34%) were males while 10 were females. The age range of the leptospirosis-confirmed population was between 18 and 94 years with an average age of 45.05 ± 16.19 years. The median duration of fever at the time of presentation to the hospital was 6 days (IQR: 2 and range: 1-16).

Twenty-five patients (14.70%) were diagnosed with AKI, with 7 patients (28.00%) classified as KDIGO stage 1, 5 patients (20.00%) in KDIGO stage 2, and 13 patients (52.00%) in KDIGO stage 3. Among the leptospirosis-confirmed patients (*n* = 79), AKI was reported in 19 patients (24.05%), of whom 12 patients (63.15%) were classified as KDIGO stage 3. Two deaths were reported in the AKI patient population where all two were confirmed as having leptospirosis. Of the two deaths reported with AKI, both patients had KDIGO stage 3. Among the leptospirosis-unconfirmed patients (*n* = 91), only 6 (6.59%) developed AKI (Figure 1).

The clinical features of the leptospirosis-confirmed patient population and leptospirosis-unconfirmed patient population were compared based on the status of AKI. Among patients with confirmed leptospirosis (*n* = 79), fever (*p* = 0.0114), icterus (0.0063), anuria/oliguria (*p* = 0.0108), and hematuria (*p* = 0.0024) were found to be significantly different between patients with AKI and non-AKI. Clinical features were not found to be significantly different between AKI and non-AKI patients in the leptospirosis-unconfirmed group (Table 1).

The patient characteristics (age and gender) and hematological/biochemical parameters of patients based on the AKI status are compared and mentioned in Table 2. When considering leptospirosis-confirmed patients and leptospirosis-unconfirmed patients, there was no significance found with the gender and status of AKI. There was no significant difference in age between patients with AKI and those without AKI. Further, when considering the leptospirosis-confirmed patient population (*n* = 79), AKI patients had high WBC counts, elevated neutrophil percentage, reduced lymphocyte percentage, high BUN levels, higher SGPT value, elevated total bilirubin and direct bilirubin levels, increased CRP levels, lower platelet levels, and high K level compared to non-AKI patients. There was no significance found in levels of SGOT and hemoglobin between AKI and non-AKI of the leptospirosis-confirmed patient population. Further, Na ion levels of AKI and non-AKI patients did not show any significant difference in the two groups.

#### 3.1.1. KIM-1 Expression

KIM-1 analysis among the patients with confirmed leptospirosis revealed a significant elevation of median serum (143.80 vs. 37.77, *p* < 0.0001) and urine (1497.00 vs. 463.60, *p* = 0.0053) KIM-1 in patients who developed AKI compared to patients without AKI. Serum KIM-1 expression was found to be 3.8-fold times higher in the leptospirosis AKI patients compared to non-AKI patients while urine KIM-1 levels showed a 3.2-fold increase. In the patients without confirmed leptospirosis, serum KIM-1 (44.94 vs. 13.71, *p* = 0.0233) showed a significant elevation among the six patients with AKI while urine KIM-1 (156.00 vs. 76.28, *p* = 0.4956) was not significantly elevated in this group (Table 3).

**Figure 2:**
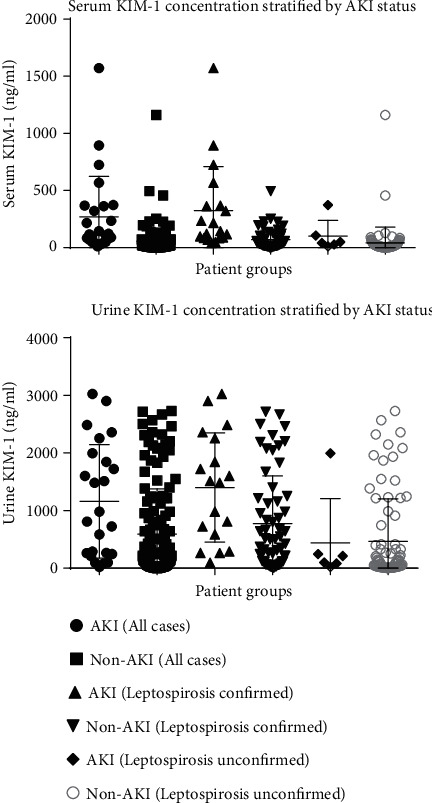
Serum and urine KIM-1 concentration stratified by AKI status.

When the expression of KIM-1 was compared between AKI patients with confirmed and unconfirmed leptospirosis, a significantly elevated serum (143.80 vs. 44.94, *p* = 0.0408) and urine (1497.00 vs. 156.00, *p* = 0.0092) KIM-1 expression was observed in patients in the confirmed leptospirosis group suggesting that KIM-1 expression could be useful in discriminating AKI patients with and without leptospirosis. Urine KIM-1 expression was found to be 9.6-fold times higher in the AKI patients with confirmed leptospirosis while serum KIM-1 levels showed a 3.2-fold increase. Furthermore, serum (37.77 vs. 13.71, *p* < 0.0001) and urine (463.60 vs. 76.28, *p* = 0.0002) KIM-1 levels were significantly raised in the leptospirosis-confirmed non-AKI group compared to the leptospirosis-unconfirmed non-AKI group (Figure 2).

#### 3.1.2. MCP-1 Expression

Among patients with confirmed leptospirosis (*n* = 79), median serum (457.30 vs. 120.90, *p* = 0.0080) and urine (450.90 vs. 133.30, *p* = 0.0019) MCP-1 were also significantly higher in patients with AKI, whereas among leptospirosis-unconfirmed patients, the findings were not significant between the two groups in the serum (132.60 vs. 94.09, *p* = 0.6178) and urine (209.50 vs. 144.60, *p* = 0.9036) (Table 3). In terms of fold difference, serum MCP-1 showed 3.8 while urine MCP-1 showed 3.4 among leptospirosis AKI patients compared to non-AKI patients.

**Figure 3:**
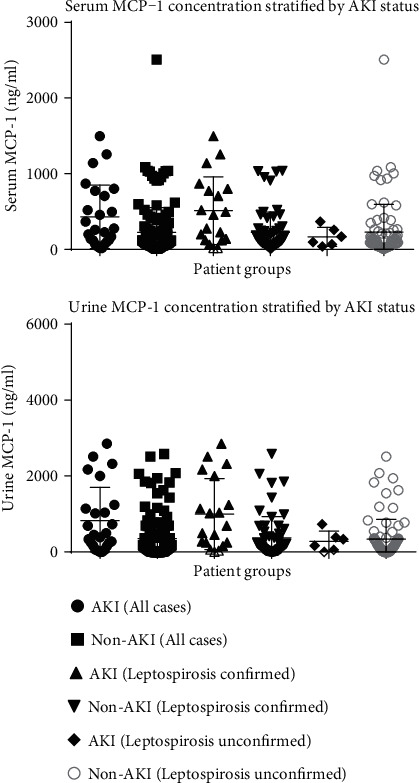
Serum and urine MCP-1 concentration stratified by AKI status.

Further, serum (457.30 vs. 132.60, *p* = 0.1004) and urine (450.90 vs. 209.50, *p* = 0.1665) MCP-1 levels between leptospirosis-confirmed AKI and leptospirosis-unconfirmed AKI were not significantly different between the two groups. There was no significant elevation in serum (120.90 vs. 94.09, *p* = 0.1869) and urine (133.30 vs. 144.60, *p* = 0.7746) MCP-1 between leptospirosis-confirmed non-AKI and leptospirosis-unconfirmed non-AKI patients (Figure 3).

### 3.2. Biomarkers for Diagnosis of AKI

**Figure 4:**
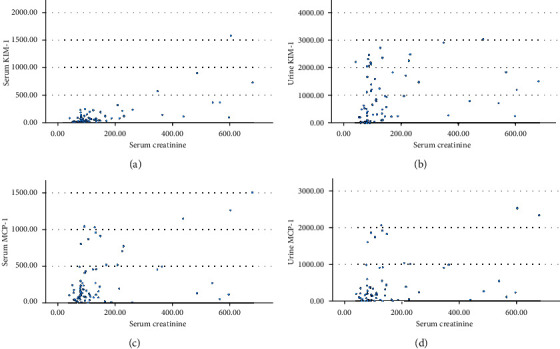
Scatter plots of (a) serum creatinine vs. serum KIM-1, (b) urine KIM-1, (c) serum MCP-1, and (d) urine MCP-1 among leptospirosis-confirmed patients.

To seek any correlation between serum creatinine and serum/urine KIM-1 and MCP-1 among leptospirosis-confirmed patients, Spearman's coefficient test was performed. The results of Spearman's coefficient test showed that levels of serum creatinine were significantly correlated with both serum (*p* < 0.000) and urine KIM-1 (*p* < 0.000) while serum (*p* = 0.002) and urine MCP-1 (*p* < 0.000) were also significantly elevated. The correlation coefficient (*ρ*) of serum and urine KIM-1 and MCP-1 was 0.594, 0.443, 0.359, and 0.401, respectively, suggesting a moderate positive correlation. The correlation between serum biomarkers and serum creatinine is shown in Figure 4.

**Figure 5:**
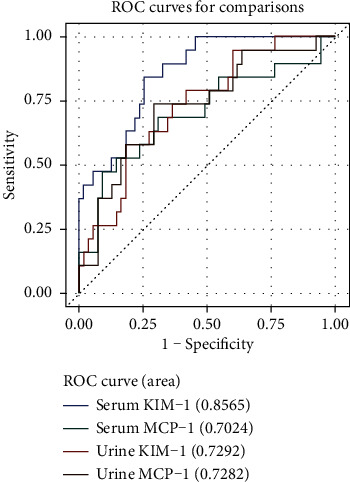
ROC curves for serum and urine KIM-1 and MCP-1 in leptospirosis-confirmed patients.

For diagnosis of AKI in leptospirosis-confirmed patients, the area under the ROC curve (AUC) for serum and urine KIM-1 was 0.8565 (95% CI 0.7689-0.9440) and 0.7292 (95% CI 0.6030-0.8554), respectively. The AUC-ROC for serum and urine MCP-1 in leptospirosis-confirmed patients gave 0.7024 (95% CI 0.5498-0.8550) and 0.7282 (95% CI 0.5925-0.8640), respectively (Figure 5). The AUC-ROC for KIM-1 and MCP-1 for the leptospirosis-unconfirmed group had values less than 0.5.

When the performed ROC DeLong AUC comparison analysis equated to serum KIM-1, there was a significant difference found between serum and urine KIM-1 (*p* = 0.0435) whereas no significant difference was found between serum KIM-1, serum MCP-1 (*p* = 0.0826), and urine MCP-1 (*p* = 0.0884).

For diagnosis of AKI, the cutoff values were determined by selecting the maximum point which gives high sensitivity and specificity. The cutoff for diagnosis of AKI among the leptospirosis-confirmed group was 82.45 ng/ml for serum KIM-1, 700.89 ng/ml for urine KIM-1, 196.48 ng/ml for serum MCP-1, and 243.58 ng/ml for urine MCP-1 (Table 4). The positive predictive value (PPV) and the negative predictive value (NPV) for each marker are mentioned in Table 4.

In the leptospirosis-confirmed patients, serum KIM-1 had the highest odds ratio (26.667) and could strongly predict AKI compared to serum MCP-1, urine MCP-1, and urine KIM-1. High WBC count (6.861) was also found to be significantly associated with AKI among the leptospirosis-confirmed patient population (Table 5).

## 4. Discussion

AKI in leptospirosis is a major complication reported in Sri Lanka [6, 8, 31]. Diagnosis of AKI in the local setting is based on serum creatinine alone. The disadvantage of AKI diagnosis based on serum creatinine is that it is elevated when 50% of renal function is compromised. Further, several other factors may influence expression of serum creatinine such as volume overload and rhabdomyolysis. Identification of reliable diagnostic biomarkers in the early detection of leptospirosis-associated AKI can considerably contribute to the initiation of early management of these patients and reduce associated morbidity and mortality [5].

Few studies attempt to investigate the value of biomarkers such as syndecan-1, intercellular adhesion molecule-1, neutrophil gelatinase-associated lipocalin, and defensin*α*1 in leptospirosis-associated AKI [5, 32, 33]. Among the novel biomarkers of renal injury, KIM-1 and MCP-1 have been suggested to be useful in the identification of AKI [22, 23]. However, the value of KIM-1 and MCP-1 in the diagnosis of AKI due to leptospirosis has not been addressed.

In this study, we found that both serum and urine KIM-1 and MCP-1 concentrations were significantly higher in patients with confirmed leptospirosis, who developed AKI compared to patients without AKI. Human KIM-1 is not measureable in healthy individuals, but is present at very high concentrations in damaged proximal tubule epithelial cells after renal injury [24]. Several studies found that KIM-1 is useful as a biomarker for the diagnosis of kidney injury [34–36]. MCP-1 has been reported to be elevated following ischemia-induced AKI [23]. A comparative study conducted on murine model-induced intrarenal injury has shown a significant increase of both MCP-1 protein and its corresponding mRNA in contrast to other novel markers such as NGAL [25, 37].

When KIM-1 and MCP-1 expression was compared between leptospirosis-confirmed and leptospirosis-unconfirmed groups, serum and urine KIM-1 levels were found to be elevated among both leptospirosis-confirmed AKI and non-AKI patients compared to the leptospirosis-unconfirmed patient population. Investigation of serum and urine MCP-1 did not show a significant difference between the leptospirosis-confirmed and leptospirosis-unconfirmed patients with AKI and without AKI. During infection, *Leptospira* enters the body and spreads into renal tissue via the hematogenous route. Within the kidney, organisms enter into peritubular capillaries, move to the interstitium and renal tubules, and finally persist in the proximal tubular lumen [32]. Damage to the proximal tubular cells results in release of KIM-1 in leptospirosis patients which explains the significantly elevated KIM-1 levels compared to MCP-1. Thus, in leptospirosis-associated AKI, KIM-1 may have more discriminatory value compared to MCP-1.

In this study, most patients presented to the hospital on the sixth day of fever. Presenting on days 5–6 is a common practice in the Sri Lankan setting. The elevated serum KIM-1 on admission was observed in patients with AKI irrespective of the day of presentation. This suggests its usefulness in identifying renal injury at any time point of the illness in this patient group. In this study, it was not possible to gather sequential data on the pattern of KIM-1 expression with time. It was not possible to collect sequential samples to evaluate the dynamics of biomarker expression over time. This is due to early discharge of patients from hospitals in the local setting where a high number of patients present for treatment. Among the patients with a confirmed diagnosis of leptospirosis who developed AKI, the ability to detect KIM-1 was observed on day 3 of fever on admission while the highest KIM-1 expression was detected on day 9 of fever on admission. Therefore, KIM-1 can be suggested to be a suitable diagnostic marker in the local setting due to its ability to predict AKI at the time of admission.

In leptospirosis cases, based on cutoff values for each biomarker (KIM-1 and MCP-1), the highest sensitivity and specificity were reported for serum KIM-1. When comparing both urine and serum specimens, KIM-1 had higher sensitivity and specificity than MCP-1. In the presence of renal assault, KIM-1 is highly upregulated in proximal tubular cells of the kidney whereas MCP-1 is produced by many cell types as a result of inflammation [34, 38]. Thus, as a biomarker in leptospirosis-mediated renal injury, KIM-1 appears to be more specific than MCP-1.

Serum KIM-1 was found to have higher diagnostic sensitivity than urine KIM-1 in these patients. The shedding of the ectodomain of KIM-1 into the tubular lumen is marked by the high concentration of KIM-1 in urine following kidney injury. Further, KIM-1 may enter the blood through several mechanisms. Research suggests that KIM-1 may be released straight into the interstitium as a result of the absence of tubular cell polarity. Further, the elevated transepithelial permeability as a result of tubular injury leads to leakage of tubular contents into the circulation. Interrupted integrity of renal microvascular endothelial cells also facilitates the KIM-1 movement into the circulation. Therefore, these studies suggest that elevated levels of KIM-1 can be detected in the blood in addition to urine and may serve as a biomarker of kidney injury. Further use of serum is useful in complications like anuria where urine samples are totally unavailable for diagnosis.

Leukocytosis is a known risk factor for AKI in leptospirosis, and in our study, leukocytosis was an independent risk factor for AKI among leptospirosis patients. Similar findings were observed by other studies where they demonstrated an independent association between leukocytosis and AKI [5, 39]. Thrombocytopenia is another known risk factor for AKI in leptospirosis that has been mentioned in several studies; however, this association was not observed in our study even though there was a significant reduction of platelet counts among leptospirosis-associated AKI [40].

Limitations of the data from this study should be recognized. In the current study, patients presented to the hospital at a median of 6 days, which did not enable the collection of early samples from the patients. In order to determine the early diagnostic utility of these biomarkers, future studies should focus on collecting specimens at the primary care level or outpatient departments to include collection of early specimens. During the study, it was not possible to collect paired serum samples from the majority of the patients. This is due to patients' recovery and discharging from the hospital prior to convalescent sample collection and not reporting to follow-up clinics after discharge. As this is a general problem when collecting paired serum, this study was limited to interpretation of MAT by using a single sample of serum. Although initially it was planned to collect sequential samples to detect KIM-1 and MCP-1 expression, there were many practical difficulties doing this. Determination of novel biomarker expression over time has given valuable data; however, it was not possible to achieve this in this study due to late presentation, delayed diagnosis, and early discharge of patients from hospitals in the local setting where a high number of patients present for treatment.

## 5. Conclusions

This study revealed that both KIM-1 and MCP-1 were elevated in leptospirosis-associated AKI and highlighted the usefulness of serum KIM-1 as a potential biomarker for detection of AKI in leptospirosis patients. The early diagnostic utility of serum KIM-1 should be investigated in future studies.

## Figures and Tables

**Table 1:** Clinical features of patients based on AKI status.

Clinical features	All cases (*n* = 170)			Confirmed diagnosis of leptospirosis (*n* = 79)			Without a confirmed diagnosis of leptospirosis (*n* = 91)		
	AKI (%) (*n* = 25)	Non-AKI (%) (*n* = 145)	*p* value^∗^	AKI (%) (*n* = 19)	Non-AKI (%) (*n* = 60)	*p* value^∗^	AKI (%) (*n* = 06)	Non-AKI (%) (*n* = 85)	*p* value^∗^
Fever	92.00	97.93	0.1062	89.47	100.00	0.0114^∗^	100.00	96.47	0.6416
Headache	48.00	66.20	0.0817	52.63	61.66	0.4875	33.33	69.41	0.0708
Myalgia	88.00	80.00	0.3461	94.73	83.33	0.2139	66.66	77.64	0.5396
Arthralgia	48.00	53.10	0.6383	42.10	55.00	0.3298	66.66	51.76	0.4823
Chills and rigors	52.00	45.51	0.5491	52.63	35.00	0.1729	50.00	52.94	0.8897
Lethargy	28.00	19.31	0.3224	26.31	20.00	0.5622	33.33	18.82	0.3911
Conjunctival suffusion	28.00	17.93	0.2411	26.31	20.00	0.5622	33.33	16.47	0.2971
Cough	28.00	38.62	0.3113	26.31	38.33	0.3429	33.33	38.82	0.7905
Dyspnea	16.00	7.58	0.1716	15.78	8.33	0.3512	16.66	7.05	0.3956
Nausea	24.00	20.68	0.7083	26.31	23.33	0.7924	16.66	18.82	0.8962
Vomiting	56.00	43.44	0.2453	52.63	45.00	0.5638	66.66	42.35	0.2489
Diarrhea	48.00	37.24	0.3094	47.36	30.00	0.1671	50.00	42.35	0.7159
Abdominal pain	20.00	31.03	0.2650	21.05	33.33	0.3135	16.66	29.41	0.5064
Icterus	32.00	13.79	0.0238^∗^	36.84	10.00	0.0063^∗^	16.66	16.47	0.9904
Anuria/oliguria	60.00	30.34	0.0041^∗^	68.42	35.00	0.0108^∗^	33.33	27.05	0.7405
Hematuria	48.00	25.51	0.0222^∗^	63.15	25.00	0.0024^∗^	0.00	25.88	0.1547

^∗^ *p* < 0.05: two-sample proportion test.

**Table 2:** Patient characteristics by AKI status.

Characteristics and hematological/biochemical parameter	All cases (*n* = 170)			Leptospirosis-confirmed patients (*n* = 79)			Leptospirosis-unconfirmed patients (*n* = 91)		
	AKI (*n* = 25)	Non-AKI (*n* = 145)	*p* value^∗^	AKI (*n* = 19)	Non-AKI (*n* = 60)	*p* value^∗^	AKI (*n* = 06)	Non-AKI (*n* = 85)	*p* value^∗^
Gender, male (%)	88.00	91.03	0.6329	89.47	86.66	0.7498	83.33	94.11	0.3067
Median									
Age (years)	50.00 (29.50)	43.00 (21.50)	0.0275^∗^	50.00 (28.00)	48.50 (22.75)	0.1799	53.50 (40.75)	42.00 (23.50)	0.3817
WBC (4‐11 × 10^3^ mm^3^)	14.20 (9.18)	9.37 (6.09)	0.0003^∗^	12.94 (7.07)	9.61 (5.85)	0.0030^∗^	16.47 (13.22)	9.35 (6.44)	0.0715
Neutrophils (%)	88.75 (10.28)	80.10 (19.69)	<0.0001^∗^	89.35 (7.08)	83.54 (20.38)	0.0024^∗^	85.32 (9.63)	78.92 (20.92)	0.1087
Lymphocytes (%)	7.02 (7.52)	11.90 (15.05)	<0.0001^∗^	4.88 (6.89)	11.45 (15.74)	0.0004^∗^	8.39 (5.16)	12.89 (13.85)	0.1874
Creatinine (60-120 *μ*mol/l)	367.7 (313.10)	98.00 (41.15)	<0.0001^∗^	443.40 (315.10)	101.60 (52.20)	<0.0001^∗^	233.30 (168.42)	94.85 (38.30)	<0.0001^∗^
BUN (2.5-6.5 mmol/l)	22.80 (17.73)	7.30 (4.95)	<0.0001^∗^	26.96 (25.26)	7.75 (6.72)	<0.0001^∗^	12.10 (11.97)	7.22 (4.45)	0.0495^∗^
SGOT (10-40 U/l)	69.00 (52.55)	47.60 (53.80)	0.0267^∗^	80.50 (53.80)	59.30 (58.17)	0.2384	52.40 (111.10)	42.00 (36.75)	0.3989
SGPT (13-31 U/l)	101.3 (119.47)	50.75 (46.57)	0.0092^∗^	106.90 (100.60)	62.50 (50.65)	0.0438^∗^	67.70 (124.00)	47.50 (31.6)	0.3107
Na^+^ (136-145 mmol/l)	133.20 (9.70)	136.0 (6.00)	0.4072	132.60 (10.9)	135.80 (8.2)	0.5106	136.50 (6.15)	136.40 (4.90)	0.8838
Total bilirubin (5-21 *μ*mol/l)	35.72 (124.94)	15.32 (25.40)	0.0087^∗^	59.24 (176.69)	13.33 (23.78)	0.0063^∗^	17.30 (19.49)	18.46 (29.77)	0.9969
Direct bilirubin (3.4 *μ*mol/l)	21.54 (85.83)	7.440 (11.03)	0.0050^∗^	34.65 (142.93)	6.33 (9.69)	0.0112^∗^	10.69 (7.92)	7.80 (11.22)	0.4573
CRP (<0.5 mg/l)	189.90 (99.90)	131.40 (141.20)	0.0003^∗^	189.90 (77.35)	157.30 (155.32)	0.0197^∗^	181.30 (153.95)	114.90 (135.20)	0.0737
Mean									
Hemoglobin (14.0-17.5 g/dl)	11.15 ± 0.42	12.19 ± 0.14	0.0098^∗^	11.10 ± 0.50	11.90 ± 0.25	0.1402	11.32 ± 0.82	12.41 ± 0.17	0.1107
Platelet (150 − 450∗10^3^/*μ*l)	79.42 ± 11.07	115.00 ± 5.25	0.0083^∗^	71.89 ± 13.75	106.3 ± 7.43	0.0277^∗^	102.0 ± 13.78	121.4 ± 7.24	0.4666
K^+^ (3.5-5.2 mmol/l)	4.71 ± 0.18	4.12 ± 0.07	0.0012^∗^	4.67 ± 0.17	4.08 ± 0.10	0.0040^∗^	4.80 ± 0.54	4.15 ± 0.10	0.0766

^∗^ *p* < 0.05: Mann-Whitney *U* test and unpaired *t*-test.

**Table 3:** Serum and urine KIM-1 and MCP-1 concentration (ng/ml) stratified by AKI status.

Renal biomarker [median, (IQR) (range)]	All cases (*n* = 170)			Leptospirosis-confirmed patients (*n* = 79)			Leptospirosis-unconfirmed patients (*n* = 91)		
	AKI (*n* = 25)	Non-AKI (*n* = 145)	*p* value^∗^	AKI (*n* = 19)	Non-AKI (*n* = 60)	*p* value^∗^	AKI (*n* = 06)	Non-AKI (*n* = 85)	*p* value^∗^
KIM-1									
Serum	115.37 (306.78) (10.75-1571.18)	21.80 (48.89) (1.18-1161.02)	<0.0001^∗^	143.80 (283.17) (40.18-1571.18)	37.77 (67.43) (2.51-493.48)	<0.0001^∗^	44.94 (149.83) (10.75-371.75)	13.71 (31.47) (1.18-1161.02)	0.0233^∗^
Urine	894.30 (1708.78) (21.50-3025.01)	180.10 (922.48) (2.56-2729.61)	0.0009^∗^	1497.00 (1771.52) (95.90-3025.01)	463.60 (1088.34) (5.03-2713.26)	0.0053^∗^	156.00 (617.49) (21.50-1996.05)	76.28 (392.40) (2.56-2729.61)	0.4956
MCP-1									
Serum	257.70 (632.33) (17.72-1496.48)	110.00 (176.44) (7.19-2508.52)	0.0053^∗^	457.30 (676.81) (17.72-1496.48)	120.90 (180.75) (7.19-1039.16)	0.0080^∗^	132.60 (224.78) (67.13-367.28)	94.09 (155.62) (7.69-2508.52)	0.6178
Urine	399.80 (933.42) (1.07-2514.43)	143.00 (318.93) (1.10-5017.05)	0.0063^∗^	450.90 (912.35) (5.67-2514.43)	133.30 (308.61) (1.19-2056.71)	0.0019^∗^	209.50 (534.10) (1.07-734.56)	144.60 (336.81) (1.10-5017.05)	0.9036

^∗^ *p* < 0.05: Mann-Whitney *U* test.

**Table 4:** Renal biomarker concentration at the best cutoff values for AKI diagnosis.

Biomarkers	Cutoff (ng/ml)	Sensitivity	Specificity	PPV	NPV
Serum KIM-1	82.45	0.789	0.750	0.894	0.633
Urine KIM-1	700.89	0.722	0.633	0.736	0.466
Serum MCP-1	196.48	0.684	0.695	0.684	0.566
Urine MCP-1	243.58	0.737	0.729	0.736	0.733

**Table 5:** Association between biomarker/clinical findings and AKI.

Biomarkers/clinical findings	Odds ratio (95% CI) unadjusted	*p* value
Serum KIM-1	26.667 (3.293-215.914)	0.002^∗^
Urine KIM-1	3.500 (0.903-13.563)	0.070
Serum MCP-1	2.400 (0.747-7.713)	0.142
Urine MCP-1	5.506 (1.677-18.074)	0.005^∗^
WBC	6.861 (1.960-24.023)	0.003^∗^
Platelet	2.500 (0.507-12.316)	0.260

## Data Availability

The datasets used and/or analyzed during the current study are available from the corresponding author on reasonable request.

## Abbreviations

AKI: Acute kidney injury
KIM-1: Kidney injury molecule-1
MCP-1: Monocyte chemoattractant protein-1
KDIGO: Kidney Disease Improving Global Outcomes
PCR: Polymerase chain reaction
MAT: Microscopic agglutination test
RIFLE: Risk, Injury, Failure, Loss, End-stage renal disease
AKIN: Acute Kidney Injury Network
EDTA: Ethylenediaminetetraacetic acid
BHT: Bed Head Ticket
ELISA: Enzyme-linked immunosorbent assay
IQR: Interquartile range
ROC: Receiver operating characteristic
AUC: Area under the curve
SPSS: Statistical Package for the Social Sciences
BUN: Blood urea nitrogen
SGOT: Serum glutamic oxaloacetic transaminase
SGPT: Serum glutamic pyruvic transaminase
CRP: C-reactive protein
WBC: White blood cells
CI: Confidence interval
PPV: Positive predictive value
NPV: Negative predictive value.
